# A study on the impact of digital infrastructure development on the health of low-income rural residents: based on panel data from 2010 to 2022

**DOI:** 10.3389/fpubh.2025.1503522

**Published:** 2025-03-12

**Authors:** Qian Wang, Zhen Ning, Meichun Tan

**Affiliations:** School of Medicine, Jiangxi University of Technology, Nanchang, Jiangxi, China

**Keywords:** digital infrastructure construction, low-income rural residents, Broadband China, multiple stage DID, China Household Tracking Survey

## Abstract

The health status of low-income rural residents is intricately linked to social equity and justice and the realization of the goal of common prosperity. Based on the CFPS data from 2010 to 2020 and the list of “Broadband China” demonstration cities, this paper employs a multi-period Difference-in-Differences (DID) approach to empirically analyze the impact of digital infrastructure construction on the health of low-income rural residents and its mechanism. The results indicate that: (1) Digital infrastructure construction had a significant positive impact on the health of rural low-income residents. After adjusting the sample period, changing the policy implementation time point, excluding other policy interference, and Propensity Score Matching-Difference-in-Differences (PSM-DID), the model was still robust. (2) The mechanism test found that digital infrastructure would improves the health of rural low-income residents through three mechanisms: the adoption of new agricultural technology, land transfer, and leisure time. (3) In terms of heterogeneity analysis, based on region type, education level, marital status and public supporting system, digital infrastructure construction can enhance the health status of low-income rural residents in the western region, those with low education level, married people and those with more public supporting system. This study suggests the continued promotion of the construction of digital infrastructure, with a focus on the construction level of the western and western regions, narrow the “digital divide” between different groups, and improve public supporting facilities in backward rural areas, so as to further enhance the health status of low-income groups.

## 1 Introduction

As a critical component of human capital, health is not only a requirement for individual holistic development but also a basic condition for economic and social development and an important symbol of national prosperity. China is predominantly an agricultural nation, with a rural population accounting for nearly 40% (1).

However, the long-term dual economic development structure has led to a significant disparity in health status between rural residents and urban residents in China (2). Compared with urban areas, low-income residents in rural areas suffer from inadequate infrastructure, poor living conditions, and insufficient access to medical services, and access to healthcare remains both challenging and costly (3). In addition, low-income residents in rural areas generally exhibit limited awareness of healthcare practices (4). These factors have significantly adversely affected the health of low-income rural residents (5). In addition, a survey of 100 villages by the Chinese Academy of Social Sciences found that 36.2% of rural households were impoverished by illness and 11.5% by disability, the first and fourth leading causes of poverty, respectively. Therefore, improving the health level of low-income rural residents is crucial for consolidate the achievements of poverty alleviation, promote the comprehensive revitalization of rural areas, and achieve common prosperity (6).

In recent years, China has been vigorously promoting the development of “Digital China” and “Cyber China.” In 2013, the State Council issued the “Broadband China Strategy and Implementation Plan,” and since 2014, it has progressively approved three batches of “Broadband China” pilot projects. As a key initiative to improve the quality and efficiency of digital infrastructure construction, many researchers have chosen to use the digital infrastructure upgrades under the “Broadband China” pilot policy as an exogenous policy shock to quantitatively analyze the effects of digital infrastructure development (7). According to a report released by the China Internet Information Center, as of the first half of 2022, China's rural digital infrastructure has been comprehensively improved, and the goal of “every village having broadband access” has been largely achieved (8). The rural Internet penetration rate increased by 1.2% compared with half a year ago and has now reached 58.8%, bringing the total number of rural internet users to 293 million. Digital infrastructure facilitates the digital transformation of rural agricultural production, residents' lives, rural governance, and other fields and has a profound impact on rural industrial transformation, farmers' employment, income generation, and other aspects (9). With the development of information technology and mass networking, the relationship between digital infrastructure and health has increasingly become a focus of traditional social science research. In foreign studies on digital infrastructure and health, research has identified a close relationship between digital inequality and population health disparities, and the digital divide is considered to be a new issue worthy of attention in health promotion and medical care. The “digital divide” refers to the inequality that exists between social groups in terms of access to, use of, and benefits from digital technologies, due to differences in the availability of information technology and internet access, as well as differences in digital literacy. Specifically, among low-income rural populations, this divide manifests as limited network coverage, imbalanced technological infrastructure, and a lack of digital skills (10). Compared with foreign research, this phenomenon has received insufficient attention in China, with research in this field emerging relatively late. Most of the relevant research in China focuses on the older adult population; the research perspective is mostly psychology, with limited consideration given to endogeneity in research methods (11, 12).

This paper builds on the aforementioned foundation and utilizes data from the 2020 China Household Tracking Survey (CFPS). It considers the policy impact of “Broadband China” as a quasi-natural experiment and employs a multi-stage DID model to explore the impact and mechanisms of digital infrastructure construction on the health of rural low-income residents at the household micro level. It further examines the heterogeneity of these effects. This analysis not only contributes to promoting the development of digital villages but also offers valuable insights into efforts to construct digital infrastructure, particularly broadband network reform, aimed at improving the health of rural low-income residents and achieving common prosperity.

The potential contributions of this paper are as follows: From a research perspective, it investigates the impact of digital infrastructure construction on the health of low-income rural residents, thereby contributing to the expansion of research on the micro-level effects of digital infrastructure in rural areas and the health of low-income populations. From a methodological standpoint, this study employs various robustness checks, including the DID model, parallel trend tests, PSM-DID, and heterogeneity treatment effect analysis, to address endogeneity and identify the causal link between digital infrastructure and health outcomes. Regarding the content of the research, this paper explores the mechanisms through which digital infrastructure development impacts the health of low-income residents, from the perspectives of agricultural machinery adoption, household income, and leisure time. The study aims to provide in-depth insights into how to effectively improve the health of rural low-income residents in the context of the digital economy, and further offers corresponding policy recommendations and improvement measures.

## 2 Literature review

Digital infrastructure has increasingly become a fundamental driver of economic transformation in rural areas (13). The existing literature largely confirms its positive impact on agricultural production, farmers' income, employment, education, and other areas in rural regions at both macro and micro levels (14–17). At a macro level, studies have shown that rural digital infrastructure construction supports the agricultural economy and production by enhancing agricultural resilience, promoting industry integration, and improving agricultural technology (18). This leads to accelerated rural economic transformation, greater economic sustainability, and improved resilience against economic shocks (19). Moreover, it facilitates the integration of rural residents into the digital society and alleviates social isolation (20). Additionally, some literature suggests that rural digital infrastructure construction can gradually narrow the urban-rural income gap (21). At the micro level, digital infrastructure construction can foster inclusive income growth, reduce poverty, and mitigate vulnerability by increasing the added value of agricultural products, creating employment opportunities, and promoting rural entrepreneurship (22). Moreover, digital infrastructure can overcome educational resource constraints, enhance social capital, and improve the return on human capital investment for children in rural areas, thereby promoting upward intergenerational income mobility (23).

Compared to other cities, the pilot cities of the “Broadband China” strategy have greatly enhanced and improved the service capacity and coverage of digital infrastructure, playing a crucial role in various aspects such as the economy, society, and people's livelihoods. The digital infrastructure construction represented by the “Broadband China” pilot policy can significantly improve the level of digital economy development in cities by enhancing urban innovation and entrepreneurship, as well as raising the total factor productivity of enterprises (24). Additionally, some scholars have examined the impact of digital infrastructure, represented by the “Broadband China” pilot policy, from the perspective of social equity. Empirical research has found that the construction of digital infrastructure helps promote upward intergenerational income mobility for rural populations, having a positive impact on achieving common prosperity (25).

The influencing factors of health have always been the core research content of health economics. The existing literature mainly analyzes the individual characteristics, employment and environment, and income gap (26). First of all, individual characteristics are the most important factors affecting health. Among them, the basic characteristics of the individual include the individual's gender, age, educational attainment, marital status, household registration, etc., and the individual family characteristics include the family size, member structure, economic status, etc (27). Some researchers used panel data from China Family Tracking Survey (CFPS) and found that adult children's migrant work would have adverse effects on the health status of the older adult, and the main reason for the deterioration of the health status of older people was the reduction in care and emotional support due to family separation (28). Secondly, employment status and community environmental characteristics also influence health. In terms of employment status, some studies took retirement age as the breakpoint and found that retirement at a normal age would have a negative impact on men's health based on the breakpoint regression model (29). In terms of community environmental characteristics, Researchers in China used the survey data of farmers and found that the relative living standard, living environment and community environment all have a significant impact on health status, that is, in addition to individual characteristics, external environment also has an important impact on health (30).

Many scholars at home and abroad have discussed the relationship between the digital infrastructure construction and health in depth, and three representative views have emerged. The first theory is health promotion theory. The health promotion model consists of three main components: cognitive-perceptual factors, modifying factors, and health-promoting actions. This model suggests that an individual's correct understanding of health knowledge, as well as environmental factors, including social relationships, interact as important predictors of health-promoting behaviors and influence the final outcome of a health-promoting lifestyle. The model supports the hypothesis that individuals with high eHealth literacy improve their health cognition by accumulating quality health information resources, thereby promoting health behaviors (31). The second theory is technology pressure theory. Digital technology pressure refers to an individual's concerns, fears, unease, and anxiety experienced when directly or indirectly exposed to, learning about, or using digital technology. This reaction may lead to psychological and physiological resistance, hindering the learning and long-term use of digital technology (32). Internet addiction is an important manifestation of technology stress, and its key feature is that technology users tend to allocate a large amount of time to internet use. Existing research has found that both internet addiction and excessive internet use, as well as reliance on social media, can increase the risks associated with internet usage (33). The third theory is indirect relationship theory, which refers to the interdependent and mutually restrictive relationship between things and phenomena through many mediating factors and intermediaries. The indirect relationship theory argues that digital technology use reflects the effect of socioeconomic status on health, and that the use of digital technology itself does not have a direct impact on health (34).

At present, most of the views tend to the health promotion theory, while the technology pressure theory actually reflects the reflection on the health risks caused by excessive use of the digital technology (35). When exploring the relationship between digital technology use and health, it is necessary not only to consider the impact of digital technology access on health but also to examine the relationship between the intensity of digital technology use and health (36).

Improvements in digital infrastructure contribute to the health of low-income farmers in three main ways: the adoption of new agricultural technologies, land transfer, and increased leisure time. GPS-controlled tractors can work around the clock, plowing, sowing, and harvesting, while collecting continuous “mobile” geographic reference data. These autonomous vehicles can perform precise operations with the help of GPS, Geographic Information Systems (GIS), and Variable Rate Technology (VRT) (37). Digitalization enables farmers to remotely control their farms and manage agricultural activities more efficiently. The development of the digital economy has significantly increased the likelihood of land transfer among farmers. Specifically, for every unit increase in the digital economy index, the probability of a household transferring land increases by 3.39%. The digital economy promotes rural land transfer by facilitating non-farm employment and entrepreneurship for farmers, as well as strengthening online social interactions and information access (38). Advanced technologies and automation systems enhance rural residents' control and flexibility in daily work, reduce the physical labor involved, and provide more leisure time, thus alleviating work-related stress (39).

In conclusion, the previous studies on the relationship between digital infrastructure construction and health, whether it is health promotion theory, technology pressure theory, or indirect relationship theory, in fact, failed to fully clarify the relationship between health and digital infrastructure construction, and there are often endogenous problems between digital infrastructure construction and health that cannot be ignored (40–42). In the further exploration of the influence mechanism, the existing research mainly forms two paths of interpersonal emotion explanation and information acquisition explanation (43). However, previous studies tend to focus on mental health, ignoring the exploration of the explanatory mechanism of information acquisition, and few empirical studies have carried out further exploration when considering more comprehensive self-rated health (44). At the same time, when discussing the relationship between socioeconomic status and health inequality from the perspective of traditional social stratification theory, health choice theory and social causality theory were mainly formed (45). However, from the traditional industrial society to the network society, the structure of social power and the flow of information have changed (46). Social stratification research needs to concentrate on the impact of information technology progress on health inequalities in the context of social change (47).

## 3 Research design

### 3.1 Specification of model

With these considerations in mind, this paper focuses on the following questions: Does the construction of digital infrastructure affect the health status of low-income rural residents? If so, what are the principal mechanisms through which the construction of digital infrastructure affects the health status of low-income rural residents? Drawing on existing research findings, this paper employs the “Broadband China” strategy as a basis for an examination of the relationship between the construction of digital infrastructure and the health of low-income rural residents. The DID model estimates the specific impact of an intervention (or policy change) on the treatment group by comparing the differences before and after the intervention between the treatment group and the control group The multi-stage Difference-in-Differences (DID) model is specified as follows:


$${\text{Health}}_{\text{it}}=\alpha +\beta_{\text{t}}{\text{Broadband}}_{\text{i}}\times {\text{Post}}_{\text{t}}+\gamma_{\text{i}}+\delta_{\text{i}}+\varepsilon_{\text{it}}$$

β_*t*_: Represents the intervention effect at different time points. *Broadband*_i_: Indicates whether an individual belongs to the intervention group. *Post*_*t*_: Indicates whether an individual is in the post-intervention period. γ_*i*:_: Individual fixed effect. δ_*t*:_: Time fixed effect, controlling for the influence at each time point. ϵ_*it*_: Error term. In Equation (1) is the dependent variable, which denotes the health status of rural low-income residents in region *i* during year *t*. Represents the dummy variable for the “Broadband China” strategy, and a set of control variables are included to account for other factors that may influence the health status of rural low-income residents. It represents the region fixed action, the year fixed action, and the random disturbance term, respectively. Is the constant term and denotes the parameter to be estimated, where represents the net policy impact on the health of low-income rural residents, with its value indicating the magnitude of the policy impact.

### 3.2 Variable selection

Table 1 presents the descriptive statistics of the variables used in this study. These are as follows:

(1) Explained variables. The explained variable in this research is the health of low-income rural people. The question “How do you assess your health status?” Respondents rated their health on a scale of 1–5, corresponding to the categories: unhealthy, fair, relatively healthy, very healthy, and extremely healthy.
(2) Core explanatory variable. The “Broadband China” policy is used as the core explanatory variable and acts as a proxy for the digital infrastructure. The “Broadband China” policy is used as the core explanatory variable and acts as a proxy for the digital infrastructure. The choice of the “Broadband China” policy as a proxy variable is mainly because it is a clear and observable policy action that effectively represents changes in digital infrastructure development. Through this policy, we can clearly track the changes before and after its implementation and link them to health impacts. Between 2014 and 2016, the establishment of “Broadband China” demonstration cities expanded incrementally, encompassing provincial counties, urban agglomerations, and prefecture-level cities and above. In this study, the “Broadband China” demonstration city dummy variable was categorized into an experimental group (coded as 1) and a control group (coded as 0). The implementation timeline of the “Broadband China” policy is divided into two stages: the years prior to the policy's implementation are coded as 0, while the implementation year and subsequent years are coded as 1. Finally, the “Broadband China” policy is represented as an interaction term between the demonstration city dummy variable and the policy implementation timeline.
(3) Control variables. The data are sourced from the GFPS (Global Family Panel Survey) database. Age: Health and age are closely related; in general, the likelihood of physical complaints increases with age. Age is used to control for the effects of physiological changes on health. Gender: Health and gender are related to physiological differences and social roles. Gender differences in health are influenced by biological factors and societal expectations. Marital status: Health and marital status are connected, as marital status can affect an individual's social support, psychological state, and potential health behaviors. Generally, as age increases, the likelihood of physical ailments also increases. Party membership: Party members in villages often bear additional administrative responsibilities, which can influence their health. Educational attainment: Higher levels of education can enhance an individual's awareness and career opportunities, thereby affecting their health. Per capita income: Higher household income allows for greater expenditure on preventing potential health risks. Public facilities: Higher levels of education can enhance an individual's awareness and career opportunities, thereby affecting their health. Per capita farmland area: A larger farmland area per capita requires more labor input, which can significantly impact health. Medical insurance: Participation in medical insurance influences health-seeking behaviors, thereby affecting overall health. Government subsidies: These subsidies provide individuals with additional financial resources to spend on disease treatment, which positively impacts health. Geographical location: resource endowment and ecological environment vary in different regions, which affect individual health.

**Table 1:** Descriptive statistics of variables.

**Variable**	**Computing method**	**Mean**	**SD**	**Min**	**Max**
Good health	Self-rated health was scored from 1 to 5	2.7423	1.4652	1	5
Broadband China	Implementation = 1; No implementation = 0	0.0528	0.2236	0	1
Age	Actual values, years	40.2711	19.4557	−8	110
Gender	1 = male; 0 = female	0.4957	0.6167	−8	1
Marital status	1 = married; 0 = unmarried	0.6111	0.4875	0	1
Party membership	1 = Party member; 0 = non-party member	0.0686	0.2527	0	1
Educational attainment	Actual values, years	7.5263	1.6668	1	16
Per capita income	Household income per capita was taken in log	9.3143	1.2998	−1.38629	15.2265
Per capita farmland area	Communal facilities	0.2405	0.8821	0	5
Per capita farmland area	Cultivated land area/total population, mu/person	1.6342	1.1452	0	7
Medical insurance	1 = yes; 0 = none	0.1595	0.3662	0	1
Whether to work	1 = yes; 0 = no	0.3601	0.3667	0	1
Government subsidies	1 = yes; 0 = none	0.3815	0.6841	0	1
Geographical location	1 = plain; 2 = hills; 3 = mountain	1.9729	0.8633	1	3
Family size	Family size	4.7288	2.0595	1	26
Agriculture	The proportion of non-farm workers in the household	0.8190	0.3647	0	1
Land circulation	Land circulation	0.4368	0.4960	0	1
Loans	Loans	0.2362	0.4248	0	1
Ease of transportation	The distance from the village committee to the county, km	1.9624	10.1201	1	130
online shopping	Whether to shop online	0.0085	0.0918	0	1
Trust in government	Trust in government	5.2235	2.8972	−16	10

### 3.3 Parallel trend tests and placebo tests

**Figure 1:**
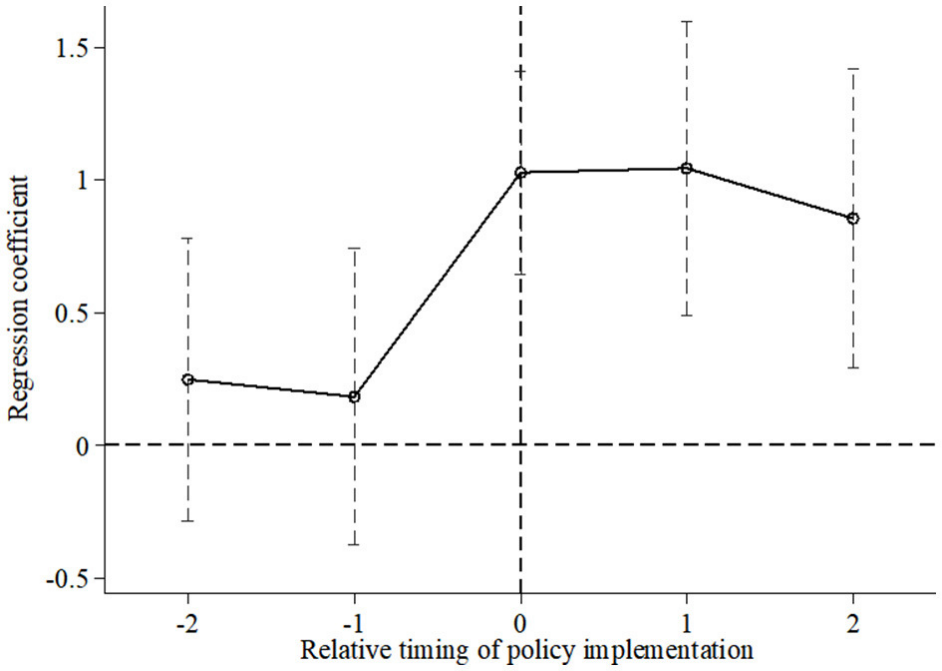
Parallel trend tests.

Figure 1 shows the results of the parallel trends tests for the model. The confidence interval for the coefficient estimate of the interaction term before 2014 includes zero, indicating no significant difference in the coefficients between the years before the policy was implemented. The results indicate that the impact of digital infrastructure construction on the health of lower-income rural dwellers has been negligible prior to the implementation of policies. The confidence intervals of the interaction term estimates are consistently above zero following the implementation of China's broadband policies. The coefficients for the health of rural low-income residents became significantly positive, with a slight upward trend in magnitude, indicating that the policy implementation indeed improved the health of these residents. Thus, these results not only confirm the hypothesis of a stable pre-treatment trend in the model but also show that policy implementation had a sustained positive effect on improving the health of low-income residents in rural areas.

**Figure 2:**
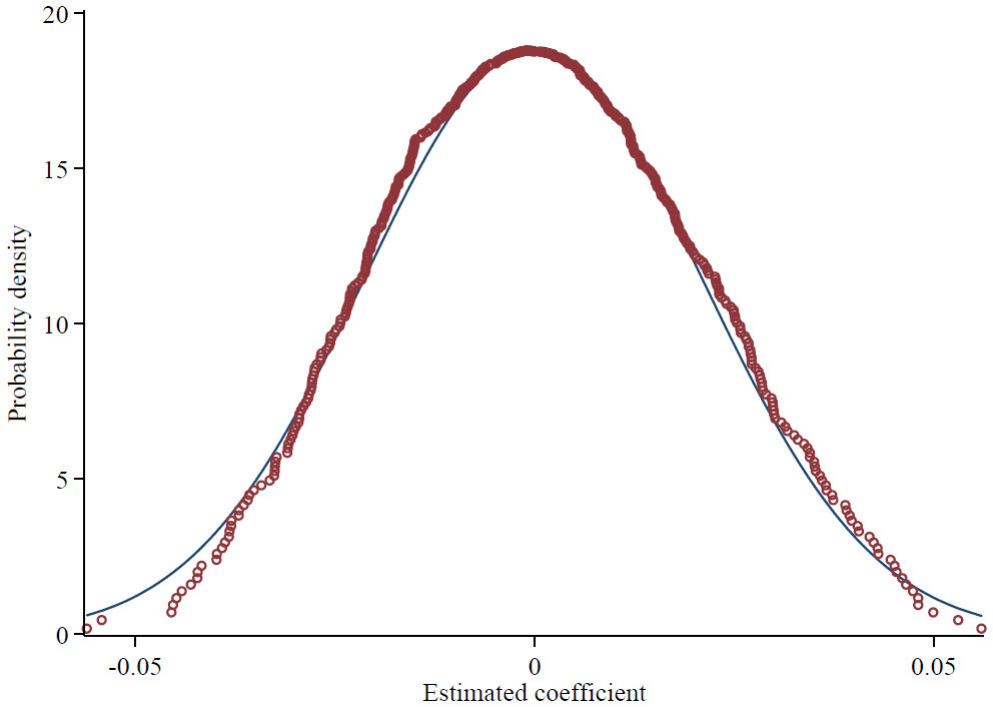
Placebo tests.

Secondly, to examine whether the estimation results are biased due to omitted variables, this paper conducts a placebo test. A placebo experiment refers to an experimental design method in which the “placebo group” does not actually receive any intervention or treatment. Instead, a hypothetical intervention scenario is simulated to test a causal relationship or validate the effectiveness of the experimental results, ensuring that potential factors or biases not considered in the experiment do not affect the outcome. Specifically, 100 counties were randomly selected from the sample and designated as a “sham” experimental group (with the distribution of selected demonstration cities consistent with the actual situation each year), while the remaining samples served as the control group. An interaction term between the placebo test dummy variable and the time variable was then constructed for regression with the estimated results shown in Figure 2. Figure 2 shows that the regression coefficient of the interaction term is close to zero, while the actual estimate of the coefficient is (0.094), represented by the vertical bar, which is clearly an outlier in the coefficient distribution of the placebo test. Therefore, there is no significant omitted variable bias in the estimated results.

### 3.4 Robustness test (adjusting the sample period, policy implementation timeline, etc.)

To test the robustness of the model estimation, different model forms or estimation methods are typically employed for verification. This paper tests robustness by adjusting the sample period, altering the policy implementation timeline, excluding other policy interferences, and employing PSM-DID. The results of the estimation process are shown in Table 2.

**Figure 3:**
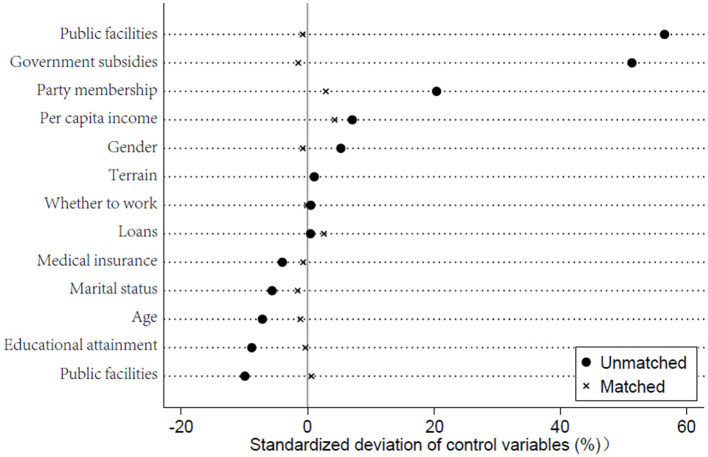
Balance tests.

1. Adjustment of the sample period. The benchmark regression uses a sample period from 2010 to 2020. To assess the model's robustness, the sample period is adjusted, and regressions are conducted using two alternative sample periods: 2010 to 2018 and 2012 to 2020. If the regression results remain significant and the coefficient signs are positive, the robustness test is considered successful. The results of the model estimation are presented in columns (1) and (2) of Table 2. The estimation results for the 2010 to 2018 period indicate that the impact of digital infrastructure construction on the health of rural low-income residents is positive and significant at the 1% level. The results of the model estimation are presented in columns (1) and (2) of Table 2. Although the estimated coefficients from these two regressions slightly differ from the benchmark regression, the direction and significance remain consistent, thereby confirming the reliability of the benchmark model.
2. Adjustment for the timing of policy implementation. The dummy variable for policy timing in this study is based on that year, as the Broadband China policy was implemented in 2014. The previous analysis confirmed that the policy implementation positively affects the health of rural low-income residents. However, factors other than digital infrastructure construction could have contributed to health improvements before 2014, or these improvements might not be directly attributable to the policy. To address this, the study uses 2012 as the new reference year for the policy timing dummy variable. If the estimation results still show a significant improvement effect, it would suggest that the observed health benefits are not attributable to digital infrastructure construction. As shown in column (3) of Table 2, the estimation of the interaction term is insignificant, indicating that there is no arbitrariness in the timing of policy implementation.
3. Exclusion of other policies. Given that the Top 100 Counties policy could influence local economic development and, in turn, affect the health of low-income rural residents, there is a possibility that the observed health improvements might primarily result from this policy rather than from digital infrastructure construction. Therefore, this study incorporates the Top 100 Counties policy into the original model to assess its impact. The aim is to determine whether the effect of the model's policy implementation remains significant. The estimation results show that when the Top 100 Counties policy is included in the model, the estimated coefficient for the demonstration policy remains significant, with its magnitude not differing significantly from the baseline regression. The coefficients for the Top 100 County policies are insignificant, indicating that these policies are not responsible for the health improvements observed among lower-income rural residents. This finding underscores the importance of the demonstration policy.
4. PSM-DID analysis. Although the previous tests addressed the assumptions of the DID method and validated the model's regression results, there may still be a self-selection bias in the experimental group. Specifically, higher-level policymakers, such as those from provincial commercial authorities, might prefer selecting cities with better development conditions as policy test points, potentially leading to biased estimation results. To address this, the PSM-DID method was reapplied to ensure robust estimation results. Following logit regression, propensity scores were obtained, and sample matching was performed accordingly. Nearest neighbor, kernel, and radius matching are common matching methods. However, a balance test must be passed to ensure the quality of matching when using PSM-DID. In this study, three matching methods—nearest neighbor, kernel, and radius—were used. The deviation between the experimental group and the control group was significantly reduced after the matching, and the samples were largely in accordance with the assumption of common support. For the sake of brevity, only the results of the balance test for the nearest neighbor matching are presented, as shown in Figure 3. The subsequent estimation of the DID after the matching produced the results shown in columns (1)–(3) of Table 3. The estimation results indicate that, regardless of the matching method used, the estimated coefficient of the model's policy interaction term remains positive and significant at the 1% level, with the coefficient size closely aligning with the benchmark regression results. This further confirms that the model estimation results in this study are robust.

**Table 2:** Baseline regression results.

**Variate**	**Cluster standard error**	**Common standard error**	**Robust standard error**	**Cluster standard error**	**Bootstrap1000**
$Policy_{i}\times {I_{t}}^{post}$	0.1120^***^	0.0964^***^	0.0964^*^	0.0964^***^	0.0784^***^
	(0.0120)	(0.0180)	(0.0450)	(0.0140)	(0.0130)
Age		−0.0056	−0.0056	−0.0056	−0.0157^***^
		(0.0070)	(0.0070)	(0.0060)	(0.0020)
Gender		0.0028	0.0028	0.0028	0.0641^***^
		(0.0120)	(0.0070)	(0.0160)	(0.0170)
Marital status		0.0815^***^	0.0815^*^	0.0815^***^	0.0005
		(0.0240)	(0.0350)	(0.0270)	(0.0310)
Party member		0.0143	0.0143	0.0143	0.0745^***^
		(0.0180)	(0.0300)	(0.0160)	(0.0170)
Degree of education		0.0063	0.0063	0.0063	0.0552^***^
		(0.0050)	(0.0160)	(0.0050)	(0.0060)
Household incomes per capita		0.0062^***^	0.0062^***^	0.0062^***^	0.0005
		(0.0010)	(0.0010)	(0.0010)	(0.0010)
Communal facilities		0.0213	0.0213^**^	0.0213	0.5754^***^
		(0.0150)	(0.0060)	(0.0170)	(0.0110)
Per capita arable land of the family		0.0903^***^	0.0903^**^	0.0903^***^	0.0903^***^
		(0.0050)	(0.0280)	(0.0050)	(0.0050)
Medical insurance		0.0597^***^	0.0597	0.0597^***^	0.1146^***^
		(0.0170)	(0.0550)	(0.0200)	(0.0200)
Whether to work		−0.5319^***^	−0.5319^***^	−0.5319^***^	−1.4389^***^
		(0.0670)	(0.0460)	(0.0850)	(0.1080)
Government subsidies		0.0210^**^	0.021	0.0210^**^	0.3175^***^
		(0.0090)	(0.0280)	(0.0100)	(0.0200)
Terrain: Hilly		−0.5409^***^	−0.5409^***^	−0.5409^***^	−0.3042^**^
		(0.1160)	(0.1200)	(0.1100)	(0.1340)
Mountain land		−0.8045^***^	−0.8045^***^	−0.8045^***^	−0.5428^***^
		(0.1540)	(0.1620)	(0.1450)	(0.1460)
Constant	9.2827^***^	9.6117^***^	9.6117^***^	9.6117^***^	10.7228^***^
	(0.0010)	(0.2750)	(0.3300)	(0.2330)	(0.1010)
R-squared	0.917	0.938	0.938	0.938	0.954

^***^, ^**^, and ^*^ indicate significance at the 1%, 5%, and 10% levels, respectively, and the numbers in parentheses are standard errors.

**Table 3:** Robustness test.

**Variate**	**Sample 2010–2018**	**Sample 2012–2020**	**Timing of policy implementation 2012**	**Exclusion of other policies**	**Kernel matching**	**Nearest neighbor matching**	**Radius matching**
	**(1)**	**(2)**	**(3)**	**(4)**	**(5)**	**(6)**	**(7)**
$Policy_{i}\times {I_{t}}^{post}$	0.1347^***^	0.0941^***^			0.1050^***^	0.1052^***^	0.0941^***^
	(0.0180)	(0.0140)			(0.0150)	(0.0151)	(0.0150)
$Policy_{i}\times {I_{2012}}^{post}$			−0.0076				
			(0.0130)				
${Policy2}_{i}\times {I_{t}}^{post}$				0.0967^***^			
				(0.0140)			
Age	−0.0122^**^	−0.0021	−0.1080^***^	−0.0063	−0.0051	−0.0052	−0.0012
	(0.0060)	(0.0060)	(0.0030)	(0.0050)	(0.0050)	(0.0051)	(0.0050)
Gender	0.0069	0.0087	0.0026^***^	0.0056	0.004	0.0043	0.007
	(0.0070)	(0.0060)	0.0000	(0.0060)	(0.0060)	(0.0061)	(0.0060)
Marital status	0.001	0.0002	0.0210^**^	0.0027	0.0115	0.0117	0.0017
	(0.0170)	(0.0160)	(0.0090)	(0.0160)	(0.0210)	(0.0212)	(0.0190)
Party membership	0.0999^***^	0.0777^***^	0.0914^***^	0.0815^***^	0.0837^***^	0.0839^***^	0.0773^***^
	(0.0310)	(0.0290)	(0.0120)	(0.0270)	(0.0280)	(0.0281)	(0.0270)
Educational attainment	0.0178	0.015	0.1502^***^	0.0143	0.0159	0.0161	0.0138
	(0.0190)	(0.0160)	(0.0150)	(0.0160)	(0.0170)	(0.0171)	(0.0170)
Per capita income	0.0002	0.0017	0.0115^***^	0.0007	0.0024	0.0025	0.0012
	(0.0050)	(0.0040)	(0.0030)	(0.0040)	(0.0040)	(0.0042)	(0.0040)
Public facilities	0.0184	0.0235^*^	0.0221^*^	0.0213	0.0037	0.0038	0.0042
	(0.0170)	(0.0120)	(0.0120)	(0.0170)	(0.0210)	(0.0211)	(0.0215)
Per capita farmland area	0.1156^***^	0.0921^***^	0.1325^***^	0.0903^***^	0.0845^***^	0.0846^***^	0.0912^***^
	(0.0070)	(0.0050)	(0.0040)	(0.0050)	(0.0060)	(0.0061)	(0.0060)
Medical insurance	0.0721^***^	0.002	0.2664^***^	0.0596^***^	0.0399^*^	0.0342^*^	0.0021
	(0.0210)	(0.0210)	(0.0170)	(0.0200)	(0.0240)	(0.0241)	(0.0210)
Whether to work	−0.5290^***^	−0.5579^***^	−0.5586^***^	−0.5319^***^	0.4937^***^	0.4939^***^	0.4946^***^
	(0.0850)	(0.0508)	(0.0580)	(0.0850)	(0.1110)	(0.1112)	(0.1102)
Government subsidies	0.0261^**^	0.0471^***^	0.1911^***^	0.0209^**^	−0.0521^***^	−0.0522^***^	−0.0462^***^
	(0.0110)	(0.0150)	(0.0090)	(0.0100)	(0.0140)	(0.0141)	(0.0140)
Terrain: Hilly	−0.4981^***^	−0.5130^***^	−0.1286^***^	−0.5390^***^	−0.5297^***^	−0.5298^***^	−0.5140^***^
	(0.1070)	(0.1120)	(0.0140)	(0.1100)	(0.1080)	(0.1081)	(0.0940)
Mountain land	−0.8178^***^	−0.7705^***^	−0.4054^***^	−0.8098^***^	−0.7859^***^	−0.7860^***^	−0.7707^***^
	(0.1510)	(0.1440)	(0.0130)	(0.1450)	(0.1510)	(0.1511)	(0.1480)
Constant	9.5080^***^	9.6333^***^	9.5249^***^	9.5994^***^	9.6875^***^	9.6876^***^	9.6917^***^
	(0.3040)	(0.2380)	(0.0220)	(0.2340)	(0.2440)	(0.2441)	(0.2400)
R-squared	0.929	0.999	0.941	0.938	0.936	0.936	0.899

^***^, ^**^, and ^*^ indicate significance at the 1%, 5%, and 10% levels, respectively, and the numbers in parentheses are standard errors.

### 3.5 Robustness test (PSM-DID analysis)

Digital infrastructure construction has been shown to significantly improve the health of low-income rural residents in the previous analysis. However, it is crucial to identify the specific channels or mechanisms through which this improvement occurs. Understanding these mechanisms is essential for guiding the formulation of effective policies to improve the health of low-income people living in rural areas. In general, there are three main channels through which the development of digital infrastructure can improve the health of low-income rural populations: the adoption of new agricultural technologies, land transfer, and increased leisure time. This study tests the following hypothesis. Given the limitations of the conventional three-step mediation method, this study adopts a two-step approach. The traditional three-step method separately tests the effects of the independent variable on the mediator, the mediator on the dependent variable, and the direct effect of the independent variable on the dependent variable. In contrast, the two-step method simplifies this by testing the interaction between the independent variable and the mediator, and its effect on the dependent variable, reducing redundant steps. It does not rely on the traditional sequential path order, making it more suitable for handling complex data and reducing model bias. This method tests only the relationship between the policy interaction term and the mechanism variables. Columns (1)–(3) of Table 4 show the results of these tests.

**Table 4:** Mechanism of action analysis.

**Variate**	**Adoption of new agricultural technologies**	**Land circulation**	**Leisure time**
	**(1)**	**(2)**	**(3)**
$Policy_{i}\times {I_{t}}^{post}$	0.0360^***^	0.0673^***^	0.0163^*^
	(0.0040)	(0.0050)	(0.0080)
Age	−0.001	−0.0171^***^	−0.0069^***^
	(0.0010)	(0.0020)	(0.0020)
Gender	0.0006	0.0008	0.0025^***^
	(0.0020)	(0.0020)	(0.0010)
Marital status	0.0111^***^	0.0121^***^	−0.0248^***^
	(0.0040)	(0.0020)	(0.0060)
Party membership	−0.0128	−0.0255^***^	0.0274^**^
	(0.0080)	(0.0100)	(0.0110)
Educational attainment	−0.0273^***^	−0.0328^***^	0.0601^***^
	(0.0050)	(0.0060)	(0.0080)
Per capita income	−0.0032^***^	0.0002	0.0076^***^
	(0.0010)	(0.0010)	(0.0020)
Public facilities	0.0113^*^	−0.0136^***^	0.0417^***^
	(0.0060)	(0.0040)	(0.0030)
Per capita farmland area	−0.0209^***^	0.0001	0.0008
	(0.0020)	(0.0020)	(0.0020)
Medical insurance	0.0103	0.0269^***^	0.0285^***^
	(0.0070)	(0.0060)	(0.0080)
Whether to work	0.1404^***^	0.1385^***^	−0.1387^***^
	(0.0180)	(0.0180)	(0.0220)
Government subsidies	0.0426^***^	0.0407^***^	−0.0459^***^
	(0.0030)	(0.0030)	(0.0030)
Terrain: Hilly	−0.1073^***^	0.2784^***^	−0.0376
	(0.0390)	(0.0610)	(0.0540)
Mountain land	−0.0916^**^	0.2638^***^	0.0376
	(0.0400)	(0.0670)	(0.0740)
Constant	0.8676^***^	0.2850^***^	0.1473^***^
	(0.0810)	(0.0910)	(0.0430)
R-squared	0.977	0.908	0.933

^***^, ^**^, and ^*^ indicate significance at the 1%, 5%, and 10% levels, respectively, and the numbers in parentheses are standard errors.

Column (1) shows that the demonstration policy has a significant positive effect on the adoption of new agricultural technology. The policy has reduced the heavy physical labor by increasing farmers' adoption of technology, thereby lowering health issues caused by excessive labor. Specifically, the application of advanced technologies not only improved production efficiency but also reduced the health risks farmers are exposed to during agricultural activities, particularly in high-intensity physical labor processes such as planting and tilling. Column (2) indicates that the policy significantly positively affects land transfer among rural low-income residents, suggesting that digital infrastructure construction encourages land transfers, thereby reducing the health risks associated with farming. Through digital platforms, farmers can access detailed information about land transfer at any time, including market prices, transfer conditions, and the credibility of the transfer parties. This not only reduces the risks associated with land transfer but also enhances farmers' enthusiasm for participating in it. Farmers can subcontract or sell part of their land, thereby reducing their farming burden, which in turn alleviates the intensity of physical labor and lowers the related health risks. Column (3) shows that the policy significantly increases leisure time for rural low-income residents, thereby contributing to improved health. The development of digital infrastructure has not only improved agricultural productivity but also enabled rural residents to reduce their working hours, allowing them more time for rest and relaxation, thereby enhancing their overall health. The increased leisure time gives farmers the opportunity to engage in more health-promoting activities, such as exercise, socializing, and relaxation, which in turn reduces health risks associated with overwork.

In summary, the empirical results confirm that the policy promotes the health of rural low-income residents through three key pathways: the adoption of new agricultural technology, land transfer, and increased leisure time.

### 3.6 Analysis of heterogeneity

Given that economic and social conditions vary across regions, this study conducted a heterogeneity analysis. Table 5 presents the analysis from two dimensions: regional economic development and education level. Developed regions typically have better infrastructure and healthcare resources, while underdeveloped regions face greater health challenges. Education level influences health awareness, health behaviors, and the adoption of digital health tools. Additionally, we considered the interaction between these two variables; for example, in developed regions, even residents with lower education levels may benefit from better infrastructure and resources. To capture this effect, we introduced interaction terms into the regression model and conducted an analysis. Columns (1)–(3) of Table 5 present the heterogeneity analysis based on regional economic development. The regions are categorized into eastern, central, and western areas, with economic development levels ranging from high to low. Columns (4) and (5) represent the heterogeneity analysis based on education level. Based on the average education level, individuals are divided into two groups: high-educated (above average) and low-educated (below average).

**Table 5:** Heterogeneity analysis: region type and educational attainment.

**Variate**	**The eastern region**	**The middle region**	**The Western region**	**Low academic qualification**	**High academic qualifications**
	**(1)**	**(2)**	**(3)**	**(4)**	**(5)**
$Policy_{i}\times {I_{t}}^{post}$	0.0083	0.0805^***^	0.1345^***^	0.1168^***^	0.0475^*^
	(0.0360)	(0.0290)	(0.0180)	(0.0340)	(0.0270)
Age	−0.0128	−0.0098	−0.0094	−0.0657^*^	−0.0888^***^
	(0.0280)	(0.0300)	(0.0260)	(0.0390)	(0.0230)
Gender	0.0085	0.0135	0.0148^*^	0.0205^***^	0.0091^***^
	(0.0090)	(0.0110)	(0.0090)	(0.0030)	(0.0020)
Marital status	0.0129	0.1901	0.0143	0.012	0.1130^***^
	(0.0260)	(0.2040)	(0.0210)	(0.0190)	(0.0220)
Party membership	0.0265	0.0696	0.1357^***^	0.0648	0.0218
	(0.0500)	(0.0520)	(0.0410)	(0.0490)	(0.0330)
Educational attainment	0.0096	0.0221^**^	0.0139^*^	—	—
	(0.0100)	(0.0110)	(0.0080)	—	—
Per capita income	0.0113^**^	0.0052	0.0078	0.0272^***^	0.0215^***^
	(0.0060)	(0.0070)	(0.0060)	(0.0070)	(0.0060)
Public facilities	0.1066^***^	0.0952^**^	0.0496^**^	0.5142^***^	0.7270^***^
	(0.0300)	(0.0430)	(0.0240)	(0.0130)	(0.0150)
Per capita farmland area	0.0846^***^	0.0908^***^	0.0902^***^	0.0839^***^	0.0827^***^
	(0.0090)	(0.0120)	(0.0090)	(0.0080)	(0.0080)
Medical insurance	0.1035^***^	0.0489	0.029	0.3820^***^	0.3350^***^
	(0.0300)	(0.0400)	(0.0340)	(0.0280)	(0.0280)
Whether to work	0.6580^***^	0.4163^**^	0.4066^***^	0.6168^***^	1.2299^***^
	(0.1290)	(0.1710)	(0.1390)	(0.1900)	(0.0870)
Government subsidies	−0.0042	−0.0456^**^	−0.0102	−0.5497^***^	−0.2860^***^
	(0.0190)	(0.0220)	(0.0150)	(0.0190)	(0.0120)
Terrain: Hilly	–	–	–	−0.1423	−0.3553^**^
	–	–	–	(0.3000)	(0.1490)
Mountain land	–	–	–	−0.5929	−0.5218^***^
	–	–	–	(0.7330)	(0.1830)
Constant	9.2995^***^	10.0099^***^	8.4704^***^	10.2439^***^	10.4071^***^
	(0.3630)	(0.4420)	(0.3470)	(0.3800)	(0.1080)
R-squared	0.953	0.915	0.912	0.934	0.928

^***^, ^**^, and ^*^ indicate significance at the 1%, 5%, and 10% levels, respectively, and the numbers in parentheses are standard errors.

The first three columns indicate that the policy improved the health of lower-income people in the east, center, and west. However, the effect is not significant in the eastern region, while it is significant at the 1% level in the central and western regions. The heterogeneity estimates suggest that the health improvement effects of building digital infrastructure are greatest in the western area, followed by the central area, and lowest in the eastern area. This may be because the eastern region already had relatively advanced digital infrastructure, limiting the policy's additional impact. This viewpoint is supported by relevant literature. For example, some studies show that the eastern region has already taken the lead in digital transformation, with significant investments from both the government and enterprises, and the results of digital infrastructure development have already become evident. Therefore, although the policy has had a positive impact on the health of low-income populations in the eastern region, its effects are relatively limited compared to those in the central and western regions.

Columns (4) and (5) show that digital infrastructure construction positively impacts the health of low-income residents, both for those with low and high education levels, with significance at the 1% and 10% levels, respectively. The estimated coefficients for educational heterogeneity suggest that the policy's impact on health is greater for low-income residents with lower education levels. This may be because less-educated individuals have limited ability to access digital information on their own, and the policy helps to compensate for this deficiency.

Table 6 presents the analysis from the perspectives of marital status and family size. Columns (1) and (2) of Table 6 analyze heterogeneity based on marital status, dividing the sample into unmarried and married individuals. Columns (3) and (4) represent heterogeneity based on the availability of public facilities. The sample is divided into areas with more or fewer public facilities, according to whether the number of public facilities is above or below the average value.

**Table 6:** Heterogeneity analysis: marital status and public matching.

**Variate**	**Unmarried**	**Married**	**Areas with few public facilities**	**Public supporting areas**
	**(1)**	**(2)**	**(3)**	**(4)**
$Policy_{i}\times {I_{t}}^{post}$	0.0605^***^	0.1107^***^	0.0915^***^	0.0991^***^
	(0.0220)	(0.0170)	(0.0350)	(0.0150)
Age	−0.008	−0.0281^***^	−0.0165	−0.0146^**^
	(0.0100)	(0.0070)	(0.0120)	(0.0060)
Gender	0.0383^**^	0.0033	0.023	0.0067
	(0.0180)	(0.0070)	(0.0200)	(0.0070)
Marital status	—	—	0.0693	0.0482
	—	—	(0.0570)	(0.0340)
Party membership	0.033	0.0053	0.0617^**^	0.0205
	(0.0330)	(0.0200)	(0.0310)	(0.0180)
Educational attainment	0.023	0.021	0.0081	0.0228
	(0.0290)	(0.0200)	(0.0380)	(0.0180)
Per capita income	0.0056	0.0028	0.0054	0.005
	(0.0070)	(0.0040)	(0.0070)	(0.0040)
Public facilities	0.0007	0.0232	—	—
	(0.0460)	(0.0190)	—	—
Per capita farmland area	0.0808^***^	0.0906^***^	0.1025^***^	0.0827^***^
	(0.0100)	(0.0070)	(0.0220)	(0.0060)
Medical insurance	0.0478	0.0550^**^	0.1071^**^	0.0635^***^
	(0.0550)	(0.0220)	(0.0460)	(0.0220)
Whether to work	0.4227^***^	0.6207^***^	1.1646^***^	0.3077^***^
	(0.1530)	(0.1270)	(0.2330)	(0.0970)
Government subsidies	−0.0106	−0.0555^***^	−0.0629^*^	0.0206^*^
	(0.0160)	(0.0170)	(0.0350)	(0.0110)
Terrain: Hilly	−0.8157^***^	−0.3023^***^	−0.3105	−0.224
	(0.2040)	(0.1150)	(0.2240)	(0.1990)
Mountain land	−1.2977^***^	−0.3242^**^	−0.4844	−0.159
	(0.2260)	(0.1360)	(0.3050)	(0.1250)
Constant	9.0472^***^	9.5426^***^	8.8943^***^	9.1862^***^
	(0.4960)	(0.2980)	(0.7780)	(0.2660)
R-squared	0.967	0.934	0.958	0.952

^***^, ^**^, and ^*^ indicate significance at the 1%, 5%, and 10% levels, respectively, and the numbers in parentheses are standard errors.

Columns (1) and (2) show that the policy has improved the health of both married and unmarried low-income residents, both of which are significant at 1% levels. The estimated coefficients for marital status suggest that the health effects of digital infrastructure are greater for married low-income residents. This may be because married individuals often spend more time and energy caring for the older adult and children in addition to working, resulting in generally poorer health compared to unmarried individuals. The communication capabilities of digital infrastructure can help reduce the time married individuals need to care for the older adult and children, thereby alleviating their stress more effectively.

Columns (3) and (4) show that the construction of digital infrastructure has a positive effect on the health of low-income residents in both areas with more public facilities and areas with fewer public facilities, and both effects are significant at the 1% level. The estimated coefficients for public facilities suggest that the health improvement effect of digital infrastructure construction is greater in areas with more public support, possibly because greater access to public facilities provides more opportunities for residents to relax and exercise, which benefits their health.

## 4 Discussion and conclusions

Using CFPS data from 2010 to 2020 and the list of “Broadband China” demonstration cities, this study selected low-income rural residents with a per capita household income of < 2,300 yuan in 2010 as the sample and empirically analyzed the impact of digital infrastructure construction on their health and its underlying mechanisms. The results showed that:

(1) Construction of digital infrastructure had a significant positive effect on the health of low-income people living in rural areas, with significance at the 1% level. Specifically, for every unit increase in digital infrastructure construction, there was a 0.0964 unit improvement in the health of low-income rural residents. Parallel trend and placebo tests confirmed the model's validity. Further robustness tests—adjusting the sample period, altering the policy implementation timeline, excluding other policy interferences, and employing PSM-DID—demonstrated that the results remained robust, confirming the reliability of the benchmark regression estimates.
(2) Regarding mechanism testing, the two-step method was used to identify the mechanisms at play. Construction of digital infrastructure was found to improve the health of low-income residents in rural areas through three main channels: the adoption of new agricultural technology, land transfer, and increased leisure time. This improvement is primarily due to the construction of digital infrastructure, which facilitates access to relevant policy information for rural residents. Furthermore, the development of information technology can improve the health of low-income populations by promoting the dissemination of modern medical knowledge and improving access to health information. This, in turn, increases the adoption of new agricultural technologies, encourages land transfer, and extends leisure time. The construction of digital infrastructure has improved the health of low-income rural residents across multiple dimensions, primarily in physical health, mental health, and health behaviors. Through the adoption of new agricultural technologies, farmers have reduced the intensity of physical labor, thereby lowering the health risks associated with agricultural activities. Digital infrastructure has also increased the possibilities for information access and social interaction, enhancing rural residents' social connections and mental health. With the increase in leisure time, rural residents have more opportunities to engage in health-promoting activities such as exercise, rest, and recreation, thereby improving health behaviors. These conclusions are based on empirical analysis using CFPS data (2010–2020) and the “Broadband China” policy, derived through a two-step mechanism test and multidimensional regression analysis. The construction of digital infrastructure has not only improved farmers' work efficiency but also enhanced their quality of life, thus having a positive impact on their health.
(3) In terms of heterogeneity analysis, digital infrastructure construction was found to improve the health of low-income rural residents across various dimensions, including region type, education level, marital status, and access to public support systems. The effects were particularly pronounced in the western region, among those with lower education levels, married individuals, and those with greater access to public support systems.

This paper has tentatively explored the mechanisms and effects of the construction of digital infrastructure on the self-rated health of residents under the condition of limited data, but there are still some shortcomings. The “Broadband China” policy, while significant as an information channel, may not be the most direct variable for exploring the underlying mechanisms. Thus, in the absence of more detailed variables related to health information use, the surrogate explanatory variables used in this study may introduce some bias into the mechanism estimation. Additionally, social classes may differ in their ability to process information, and the quality of online health information can have varying impacts on users' health. However, as an exploratory study of the relationship between building digital infrastructure and health in the context of the information society, this paper retains theoretical value and offers important insights. With more comprehensive survey data, future research can provide more detailed discussions and analyses.

This study has not sufficiently considered the different stages of the digital infrastructure construction lifecycle. Future research can further explore the varying impacts of each stage on the health of low-income rural residents, and provide more detailed policy recommendations based on the characteristics of each stage. For example, in the planning and construction phase, the government should conduct targeted planning based on regional characteristics, focusing on the quality and balance of network coverage, to ensure equal access to information and health management opportunities for rural residents, especially in the western and central regions. In the operational phase, attention should be given to the maintenance and upgrading of facilities to ensure long-term sustainability. In the upgrading phase, the synchronization of technological updates should be prioritized to enhance the accessibility and quality of health services. In conjunction with education system reforms, particularly targeting low-education groups, digital literacy training should be implemented to help them better use information technology to improve personal health.

## Data Availability

The raw data supporting the conclusions of this article will be made available by the authors, without undue reservation.

## References

[1] Hu Z Li Y Long H Kang C. The evolution of China's rural depopulation pattern and its influencing factors from 2000 to 2020. Appl Geograp. (2023) 159:103089. 10.1016/j.apgeog.2023.103089

[2] Wang X Shao S Li L. Agricultural inputs, urbanization, and urban-rural income disparity: evidence from China. China Econ Rev. (2019) 55:67–84. 10.1016/j.chieco.2019.03.009

[3] Shi L. Health care in China: a rural-urban comparison after the socioeconomic reforms. Bull World Health Organ. (1993) 71:723. 8313490 PMC2393531

[4] Wang L Wang Z Ma Q Fang G Yang J. The development and reform of public health in China from 1949 to 2019. Global Health. (2019) 15:1–21. 10.1186/s12992-019-0486-6 31266514 PMC6604346

[5] Zhao Z. Income inequality, unequal health care access, and mortality in China. Popul Dev Rev. (2006) 1, 461–83. 10.1111/j.1728-4457.2006.00133.x

[6] Guo H Yang Y Pan C Xu S Yan N Lei Q. Study on the impact of income gap on health level of rural residents in China. Int J Environ Res Public Health. (2022) 19:7590. 10.3390/ijerph19137590 35805243 PMC9265866

[7] Huang Z Tao Y Zhang Q. The road to entrepreneurship: the effect of China's broadband infrastructure construction. Econ Anal Policy. (2023) 80:1831–47. 10.1016/j.eap.2023.11.004

[8] Ren Y. Rural China staggering toward the digital era: evolution and restructuring. Land. (2023) 12:1416. 10.3390/land12071416

[9] Bi J. Can rural areas in China be revitalized by digitization? A dual perspective on digital infrastructure and digital finance. Fin Res Lett. (2024) 67:105753. 10.1016/j.frl.2024.105753

[10] Lythreatis S Singh SK El-Kassar AN. The digital divide: a review and future research agenda. Technol Forecast Soc Change. (2022) 175:121359. 10.1016/j.techfore.2021.121359

[11] Niu S. The impact of the digital divide on the mental health of older people: SHS web of conferences. EDP Sci. (01010) 2023:157. 10.1051/shsconf/202315701010

[12] Sun K Zhou J. Understanding the impacts of internet use on senior citizens' social participation in China: evidence from longitudinal panel data. Telemat Inform. (2021) 59:101566. 10.1016/j.tele.2021.101566

[13] Ydyrys S Ibrayeva N Abugaliyeva F. Regulatory and legal support for the development of digital infrastructure in rural areas as a factor in improving the level of sustainable development and quality of life of the rural population. J Environ Manage Tour. (2023) 14:2271–80. 10.14505/jemt.v14.5(69).08

[14] Han H Hai C Wu T Zhou N. How does digital infrastructure affect residents' healthcare expenditures? Evidence from Chinese microdata. Front Public Health. (2023). 11:1122718.1. 10.3389/fpubh.2023.1122718 37213630 PMC10192711

[15] Chen Y Zhang L Wei M. How does smart healthcare service affect resident health in the digital age? Empirical evidence from 105 cities of China. Front Public Health. (2022) 9:833687.2. 10.3389/fpubh.2022.1085256 35127633 PMC8813850

[16] You ZT Zhong M Gao Q Wei HX Zeng XH. The impact of digital economy on residents' health: based on the perspective of population ageing. Front Public Health (2021) 9:725971. 10.3389/fpubh.2021.725971 34381758 PMC8350039

[17] Seshadri DR Davies EV Harlow ER Hsu JJ Knighton SC Walker TA Drummond CK. Wearable sensors for COVID-19: a call to action to harness our digital infrastructure for remote patient monitoring and virtual assessments. Front Digital Health. (2020) 2:8. 10.3389/fdgth.2020.00008 34713021 PMC8521919

[18] Jian L. Digital infrastructure construction and Chinese agricultural and rural modernization: based on the mediating effect of rural industrial diversification development and digital literacy. Econ Surv. (2024) 41:3. 10.15931/j.cnki.1006-1096.2024.03.011

[19] Hui CY Abdulla A Ahmed Z Goel H Habib GMM Hock TT. Mapping national information and communication technology (ICT) infrastructure to the requirements of potential digital health interventions in low-and middle-income countries. J Global Health. (2022) 12:04094. 10.7189/jogh.12.04094 36579436 PMC9804211

[20] Ashmore FH Farrington JH Skerratt S. Community-led broadband in rural digital infrastructure development: Implications for resilience. J Rural Stud. (2017) 54:408–25. 10.1016/j.jrurstud.2016.09.004

[21] Li X He P Liao H Liu J Chen L. Does network infrastructure construction reduce urban–rural income inequality? Based on the “Broadband China” policy. Technol Forecast Soc Change. (2024) 205:123486. 10.1016/j.techfore.2024.123486

[22] Mefi NP Asoba SN. A digital infrastructure perspective for accelerated rural entrepreneurship. Contemp Res Bus Manage Econ. (2024) 8, 84–92. 10.9734/bpi/crbme/v8/8109E

[23] Jia H. Impact of digital infrastructure construction on the migrants' utilization of basic public health services in China. BMC Health Serv Res. (2024) 24:761. 10.1186/s12913-024-11221-7 38910262 PMC11194986

[24] Hu ZH Yang M Wang MY. Research on the impact of network infrastructure construction on the development of urban digital economy: Based on the quasi-natural experiment of the “Broadband China” policy. J Jilin Bus Technol Coll. (2024) 40:5–13. 10.19520/j.cnki.issn1674-3288.2024.06.008

[25] Peng J Zhao XL. The impact of digital economy development on intergenerational occupational mobility: A fairness perspective in the context of Chinese-style modernization. Mod Financ Econ. (2024) 44:23–41. 10.19559/j.cnki.12-1387.2024.07.001

[26] Kim H S Lee E Y. Narrowing the digital infrastructure divide among cities and rural areas. In: 2010 The 12th International Conference on Advanced Communication Technology (ICACT), vol. 1. New York City: IEEE (2010). p. 71–4.

[27] Forero Bucheli M. Rural education in Peru: a study of its performance, physical and digital infrastructure, gender, linguistic, and social and cultural development [Master of Liberal Studies Theses] (2023), 106.

[28] Song Q. Aging and separation from children: the health implications of adult migration for elderly parents in rural China. Demogr Res. (2017) 2017:37. 10.4054/DemRes.2017.37.55 30581322 PMC6301042

[29] Li J Yuan B Lan J. The influence of late retirement on health outcomes among older adults in the policy context of delayed retirement initiative: an empirical attempt of clarifying identification bias. Arch Public Health. (2021) 79:59. 10.1186/s13690-021-00582-8 33902694 PMC8077823

[30] Nimrod G. Technostress: measuring a new threat to well-being in later life. Aging Ment Health. (2018) 22:1086–93. 10.1080/13607863.2017.1334037 28562064

[31] Cragg L Davies M Macdowall W. Health promotion theory. New York City: McGraw-Hill Education (2013).

[32] Ayyagari R Grover V Purvis R. Technostress: Technological antecedents and implications. MIS Q. (2011) 35, 831–58. 10.2307/41409963

[33] Shu Q Tu Q Wang K. The impact of computer self-efficacy and technology dependence on computer-related technostress: a social cognitive theory perspective. Int J Hum Comput Interact. (2011) 27:923–39. 10.1080/10447318.2011.555313

[34] Glymour MM Avendano M Kawachi I. Socioeconomic status and health. Soc Epidemiol. (2014) 2:17–63. 10.1093/med/9780195377903.003.0002

[35] Lee YK Chang CT Lin Y Cheng ZH. The dark side of smartphone usage: Psychological traits, compulsive behavior and technostress. Comput Human Behav. (2014) 31:373–83. 10.1016/j.chb.2013.10.047

[36] Zhu Y Zhou Y Long C Yi C. The relationship between internet use and health among older adults in China: the mediating role of social capital. In: Healthcare, vol. 9. Basel: MDPI. (2021). p. 559. 10.3390/healthcare9050559 PMC8151524 34068702

[37] Klerkx L Jakku E. Labarthe P. A review of social science on digital agriculture, smart farming and agriculture 40: New contributions and a future research agenda. Wageningen J Life Sci. (2019) 90:100315. 10.1016/j.njas.2019.100315

[38] Zheng P Li Y Li X. The impact of the digital economy on land transfer-out decisions among Chinese farmers: Evidence from CHFS micro-data. Sci Rep. (2024) 14:19684. 10.1038/s41598-024-70605-1 39181923 PMC11344833

[39] Inoue K. Changes of working and sleeping hours of farmers in accordance with technological innovation in agriculture[J]. J Hum Ergol. (1974) 3:131–41. 10.11183/jhe1972.3.131 4465402

[40] Schram A Friel S Freeman T Fisher M Baum F. Digital infrastructure as a determinant of health equity: an Australian case study of the implementation of the National Broadband Network. Austr J Public Admin. (2018) 77:829–42. 10.1111/1467-8500.12323

[41] Liu Y Liu K Zhang X Guo Q. Does digital infrastructure improve public Health? A quasi-natural experiment based on China's Broadband policy. Soc Sci Med. (2024) 344:116624. 10.1016/j.socscimed.2024.116624 38290184

[42] Ramnath VR. Global telehealth and digital health: How to support programs and infrastructure. In: Emerging Practices in Telehealth (2023). Cambridge: Academic Press. p. 163-182.

[43] Burleson BR. The experience and effects of emotional support: what the study of cultural and gender differences can tell us about close relationships, emotion, and interpersonal communication. Pers Relatsh. (2003) 10:1–23. 10.1111/1475-6811.00033

[44] Ingram L. Question of Economic Inequality and Subjective Wellbeing: Happiness, Mental Health, and Political Inequality Perspective. Strasbourg: Université de Strasbourg (2021).

[45] Øversveen E Rydland HT Bambra C Eikemo TA. Rethinking the relationship between socio-economic status and health: Making the case for sociological theory in health inequality research. Scand J Public Health. (2017) 45:103–12. 10.1177/1403494816686711 28078944

[46] Castells M. Informationalism, networks, and the network society: a theoretical blueprint. Netw Soc Cross Cult Perspect. (2004) 3–45. 10.4337/9781845421663.00010

[47] Yu J Meng S. Impacts of the internet on health inequality and healthcare access: a cross-country study. Front Publ Health. (2022) 10:935608. 10.3389/fpubh.2022.935608 35757602 PMC9218541

